# Dibenzothiophene Removal from Fuel Oil by Metal-Organic Frameworks: Performance and Kinetics

**DOI:** 10.3390/ijerph20021028

**Published:** 2023-01-06

**Authors:** Han Chen, Zhipeng Huang, Juping You, Yinfeng Xia, Jiexu Ye, Jingkai Zhao, Shihan Zhang

**Affiliations:** 1 Key Laboratory for Technology in Rural Water Management of Zhejiang Province, Zhejiang University of Water Resources and Electric Power, Hangzhou 310018, China; 2 Key Laboratory of Microbial Technology for Industrial Pollution Control of Zhejiang Province, College of Environment, Zhejiang University of Technology, Hangzhou 310014, China; 3 School of Petrochemical Engineering & Environment, Zhejiang Ocean University, Zhoushan 316022, China

**Keywords:** MOFs, DBT, adsorptive desulfurization, kinetics, Cu-ABTC

## Abstract

Desulfurization of organic sulfur in the fuel oil is essential to cut down the emission of sulfur dioxide, which is a major precursor of the acid rain and PM_2.5_. Currently, hydrodesulfurization is regarded as a state-of-art technology for the desulfurization of fuel oil. However, due to the stringent legislation of the fuel oil, the deep desulfurization technology is urgent to be developed. Adsorptive desulfurization method is promising due to the high selectivity and easy operation. The development of efficient adsorbent is important to advance this technology into industrial application. In this work, the five types of metal-organic frameworks (MOFs), including Cu-BTC, UMCM-150, MIL-101(Cr), UIO-66, and Cu-ABTC were synthesized for the adsorption of dibenzothiophene (DBT), a typical organic sulfur compound in the fuel oil. The experimental results revealed that the adsorption capacity of the five MOFs followed the order of Cu-ABTC, UMCM-150, Cu-BTC, MIL-101(Cr), and UIO-66, which adsorption capacities were 46.2, 34.2, 28.3, 26.3, and 22.0 mgS/g, respectively. The three types of Cu-based MOFs such as Cu-ABTC, UMCM-150, and Cu-BTC outperformed the Cr-based MOFs, MIL-101, and Zr-based MOFs, UIO-66. Since the surface area and pore volumes of the Cu-based MOFs were not the greatest among the tested five MOFs, the physical properties of the MOFs were not the only limited factor for the DBT adsorption. The π-complexation between DBT and linkers/metal in the MOFs was also important. Kinetic analysis showed that the DBT adsorption onto the five tested MOFs follows the pseudo-second-order kinetics, confirming that the chemical π-complexation was also contributed to the DBT adsorption. Furthermore, the operation parameters such as oil-adsorbent ratio, initial sulfur concentration and adsorption temperature for the DBT adsorption onto Cu-ABTC were optimized to be 100:1 g/g, 1000 mgS/L and 30 °C, respectively. This work can provide some insights into the development of efficient adsorbent for the organic sulfur adsorption.

## 1. Introduction

The organic sulfur contaminants in fuel oil, such as dibenzothiophenes (DBT), benzothiophene (BT), thiophenes (TH), mercaptans, and their derivatives, generate sulfur dioxides (SO_2_) after combustion. SO_2_ is a main precursor of the acid rain and PM_2.5_, which causes soil acidification, building corrosion, and other ecosystems damages [1,2,3]. Moreover, exposure to SO_2_ also causes respiratory illnesses, trigger asthma, and aggravate heart disease [4]. Therefore, stringent sulfur content limitation of fuel oil was set by China and European Union [5]. The research on efficient organic sulfur compounds removal from fuel oil is urgent to the oil refining industry.

The technology of hydrodesulfurization (HDS) is mature and intensively used to remove organic sulfur from the fuel oil. Typically, the HDS was operated at high temperatures of 300–450 °C and high pressures of 3.0–5.0 MPa with an intensive consumption of hydrogen gas [1,6], resulting in a high cost of HDS. Therefore, biodesulfurization, extractive desulfurization, oxidative desulfurization, and adsorptive desulfurization have been proposed for the deep desulfurization of the fuel oil [7,8,9,10]. The thiophenic sulfur in the fuel oil such as DBT is a key pharmaceutical intermediate and its derivatives are widely used as agrochemicals, dyes, photoactive compounds, liquid crystals, and conducting polymers [10]. Therefore, it is valued to recovery thiophenic sulfur from fuel oil. Adsorptive desulfurization is a promising technology to recover the organic sulfur due to its high selectivity, low cost, and mild operation conditions. Typically, its removal efficiency depends upon the chemical-physical properties of adsorbent, such as pore volume, surface area, functional groups, and surface acidity [11].

Among the various adsorbents, metal-organic frameworks (MOFs) and MOFs-based materials exhibit outstanding desulfurization performance owing to its tunable pore size, large surface area, and adjustable functionalities [12,13,14,15]. Typically, MOFs consist of metal nodes or metal clusters and polyfunctional organic linkers [16]. Organic linkers act as bridging ligands between the metal atoms to form highly ordered porous three-dimensional networks [17]. Five microporous coordination polymers, such as MOF-5, HKUST-1, MOF-177, UMCM-150 and MOF-505, were synthesized to adsorb the thiophenic sulfur such as DBT, Benzothiophene (BT), and dimethyl-dibenzothiophen (DMDBT) from the simulated fuel oil [17]. Nazmul et al. developed three Cu-impregnation MOFs and found that the adsorption capacity of BT was increased by 14% when the mass ratio of Cu/Fe was 0.07 due to π-complexation between Cu and BT [18]. Huang et al. also proved that the strong binding between the immobilized Ag(I) sites and thiophene derivatives caused a high desulfurization efficiency [19]. Fan et al. loaded Mo(CO)_6_ onto MOF-5 and evaluated its adsorption performance of DBT from naphthalene, ioctane, and/or benzene, finding that the obtained Mo(CO)_6_-MOF-5 possessed relatively high affinities and selectivity for the DBT adsorption from the transportation fuels [20]. A novel heteropolyacid supported MOF fibers was synthesized and a desulfurization efficiency of 99.23% was obtained. The desulfurization efficiency was negligible decreased even after 10 times usage [21]. Mguni et al. reported that the modulated synthesis of Ni-BDC using formic acid increased the the overall adsorptive capacity by two folds [22]. The UIO-66 is regarded as a promising adsorbent due to its short synthesis time, low synthesis temperature, and massive preparation [23]. Moreover, MOFs-based materials as heterogeneous catalysts are also widely reported for the oxidative desulfurization. UiO-67 was used as packing materials in plasma discharge and improved the plasma generation due to its porous nature, which favors the formation of filamentary micro discharges and surface discharges. The conversion of CH_4_ and CO_2_ was increased by about 18% and 10%, respectively [24]. Wang et al. proposed a one-step approach to synthesize oriented ZIF-L membranes, which exhibited high H_2_ permeance in a steam reforming process [25]. Although some achievements of the thiophene sulfur removal by MOFs has been observed, efficient adsorptive desulfurization by MOFs still faces challenges. Most of the reported adsorptive desulfurization focused on the enhancement of the adsorption capacity. However, the adsorption mechanism and the role of the metal centers and organic linkers in the MOFs was barely investigated. The affinity between the metal centers in MOFs and DBT via the π-complexation may be also important for the adsorptive desulfurization.

In this work, five MOFs, such as Cu-BTC, UMCM-150, MIL-101(Cr), UIO-66, and Cu-ABTC, were synthesized and characterized by X-ray diffraction (XRD), Fourier transform infrared spectroscopy (FTIR) and Brunauer–Emmett–Teller (BET) analysis. Their adsorption performance and kinetics were investigated with various sulfur concentrations of DBT as model compound. Furthermore, the operation conditions such as oil-adsorbent ratio, temperature, adsorption time, and initial sulfur concentration were optimized. This work will provide some guidance to the design of a high-efficient and low-cost MOFs for the desulfurization of the fuel oil.

## 2. Materials and Methods

### 2.1. Reagents

DBT (C_12_H_8_S, ≥99%), H_3_BHTC (C_15_H_10_O_6_, 99.8%), H_4_ABTC (C_15_H_10_O_6_, 99.8%), ZrCl_4_ (≥99.9%), *p*-phthalic acid (C_8_H_6_O_4_, 99%), 1,4-dioxane (C_4_H_8_O_2_, 99.5%), Cu(NO_3_)_2_·2.5H_2_O (98%), Cu(NO_3_)_2_·3H_2_O (99.9%), and Cr(NO_3_)_3_·9H_2_O (99%) were purchased from Aladdin (Shanghai, China). H_3_BTC acid (C_9_H_6_O_6_, 95%) was obtained from Sigma-Aldrich (Shanghai, China). Methanol (CH_4_O, 99.5%), ethanol (CH_3_CH_2_OH, 99.8%), DMF (C_3_H_7_NO, ≥99.5%), and acetic acid (C_2_H_4_O_2_, 99%) were obtained from Yien (Shanghai, China). Octane (C_8_H_18,_99%) and HNO_3_ (65–68%) were purchased from Macklin Reagents Ltd. (Shanghai, China).

### 2.2. Synthesis of the Five MOFs

For the Cu-BTC synthesis, 1.925 g Cu(NO_3_)_2_·2.5H_2_O was dissolved in 17 mL of DI water to form solution A. 1.000 g H_3_BTC was dissolved in 34 mL of the DMF and ethanol (1:1, *v*/*v*) mixture to obtain solution B. The solutions A and B were mixed and ultrasonic treated for 20 min and then transferred to a 100 mL Teflon-lined stainless steel autoclave at 100 °C for 10 h. For the UMCM-150 preparation, 0.05 g H_3_BHTC and 0.098 g Cu(NO_3_)_2_·2.5H_2_O were dissolved in 15 mL of the DMF-dioxane-H_2_O (4:1:1, *v*/*v*) mixture and then transferred to a 100 mL Teflon-lined stainless steel autoclave at 85 °C for 6 h. The MIL-101(Cr) was obtained by mixing 2.0 g Cr(NO_3_)_3_·9H_2_O and 0.83 g *p*-phthalic acid in 20 mL DI water for the 10 min sonication and 20 min stirring. The resulted solution was transferred to a 100 mL Teflon-lined stainless steel autoclave at 218 °C for 18 h. As for the UIO-66, 0.19 g ZrCl_4_ and 0.133 g *p*-phthalic acid were dissolved in 82 mL DMF and then 4.8 g acetic acid was added. After the 20 min sonication and 20 min stirring, the mixture was transferred to a 100 mL Teflon-lined stainless steel autoclave at 120 °C for 24 h. The Cu-ABTC was synthesized by adding 0.18 g H_4_ABTC and 0.24 g Cu(NO_3_)_2_·3H_2_O into the 45mL DMF-ethanol-H_2_O (5:3:1, *v*/*v*) mixture. Then, the 2 mol/L nitric acid was added dropwise at a rate of 2 drops/sec for 5 s under stirring, and finally transferred to a 100 mL Teflon-lined stainless steel autoclave at 60 °C for 48 h. All the resulted solid-liquid mixture of the five MOFs, such as Cu-BTC, UMCM-150, MIL-101(Cr), UIO-66, and Cu-ABTC, were centrifuged and successively washed by DMF and methanol for 2 h (at least 3 times). The five MOFs were dried at 150 °C for 6 h and stored in room temperature for further usage.

### 2.3. DBT Adsorption Procedure

The adsorption performance of the five synthesized MOFs was evaluated in a thermostatic shaker using 2 mL serum bottle. In a typical test, 10 mg of each MOFs was added into 1.42 mL simulated oil with various sulfur concentrations (100–1500 mg S/L) at 30 °C and adsorption process was operated for 7 h. The samples were collected at 10, 20, 40, 60, 120, 240, 360, and 420 min, respectively, and filtered by 0.22 μm filter for further GC analysis. To optimize the adsorption conditions, the Cu-ABTC with a high adsorption capacity was selected as the representative MOFs. The influence of initial sulfur concentrations (100–3000 mg S/L), oil-adsorbent ratios (60–120 g/g), and operation temperatures (15–40 °C) on the adsorption performance were investigated. After sampling, the MOFs and simulated oil mixture was filtered and separated by 0.22 μm filter. The remained solvent was analyzed by the gas chromatograph (Shimadzu, GC-2014, Japan) equipped with the hydrogen flame ionization detector (FID). The concentration of sulfur in simulated oil is calculated as follow:

(1) $$C_{e}=m\cdot M_{S}\cdot 1000/M\cdot V$$

where *m* (g) is the mass of DBT; $M_{s}$ and *M* (g/mol) are the molecular weights of elemental sulfur and DBT, respectively; *V* (L) represents the volume of simulated oil.

The kinetics of DBT adsorption onto Cu-BTC, UMCM-150, MIL-101(Cr), UIO-66, and Cu-ABTC were analyzed by the pseudo-first-order and pseudo-second-order models [26].

(2) $$\ln(q_{e}-q_{t})=\ln q_{e}-k_{1}t$$

(3) $$\frac{t}{q_{t}}=\frac{t}{q_{e}}+\frac{1}{k_{2}q_{e}^{2}}$$

where $q_{e}$ and $q_{t}$ (mg S/g) are adsorption quantities at equilibrium and sampling time *t*; $k_{1}$ (min^−1^) is the adsorption rate constant of pseudo-first-order model and $k_{2}$ [g/(mg S·min)] is the adsorption rate constant of pseudo-second-order model; *t* presents the sampling time, s.

### 2.4. Characterization and Analytical Method

The crystallinity of the five MOFs was qualitatively analyzed by XRD (Empyrean, PANlytical B.V., Heracles Almelo, The Netherlands) with primary monochromatic high intensity Cu-Kα (λ = 0.154056 nm) at a scanning rate of 0.02°/min. The tube voltage and current were 45 kV and 40 mA, respectively. The average crystallite size of the five MOFs were calculated by the Scherrer equation in JADE. FTIR measurements were carried out using a Thermo Scientifific Nicolet iN10 (Waltham, MA, USA). The five MOFs was vacuum dried at 150 °C for 2 h. The resulted MOFs was mixed with KBr and compressed to tablets. These samples were characterized under 4000–400 cm^−1^. The surface area and pore size of the five MOFs were measured by N_2_-temperature programmed desorption at 77 K by ASAP 2460 (Micromeritics Instrument, Norcross, GA, USA). The samples were degassed under vacuum at 150 °C for 6 h before test. The specific surface area was determined based on the BJH model. The pore size distribution was analyzed by the NL-DFT model.

The concentration of sulfur in simulated oil was measured by gas chromatograph equipped with capillary column (RTX-1, 30 m × 0.32 mm × 0.25 μm), the temperature of injection, column, and FID detector were 290, 260, and 260 °C, respectively. N_2_ was used as the carrier gas at a flow rate of 24 mL/min. The volume of the injected sample was 0.6 μL. The adsorbed capacity ($q_{e}$, mg S/g) was calculated as:

(4) $$q_{e}=\left(C_{0}-C_{e}\right)\cdot V/m$$

where $C_{0}$ and $C_{e}$ are the initial and equilibrium concentrations (mg S/g), respectively; *V* is the volume of solution (mL) and *m* is the mass of MOFs (mg).

The adsorption thermodynamics of Cu-ABTC was determined based on the following equations:

(5) $$K_{d}=\frac{q_{e}}{C_{e}}$$

(6) $$\Delta G^{o}=-RTlnK_{d}$$

(7) $$lnK_{d}=-\frac{\Delta G^{o}}{RT}=-\frac{\Delta H^{o}}{RT}+\frac{\Delta S^{o}}{R}$$

where $K_{d}$ is the distribution coefficient, $\Delta G^{o}$ is Gibbs free energy (KJ/mol), $\Delta H^{o}$ is enthalpy (KJ/mol), $\Delta S^{o}$ is entropy (J/mol·K), T is the temperature (K), R is the constant of 8.314 J/(mol·K). The values of $\Delta H^{o}$ and $\Delta S^{o}$ were calculated based on the intercept and slope of the Van’t Hoff plot of $lnK_{d}$ versus 1/T, respectively.

## 3. Results and Discussion

### 3.1. Characterization of Five MOFs

**Figure 1:**
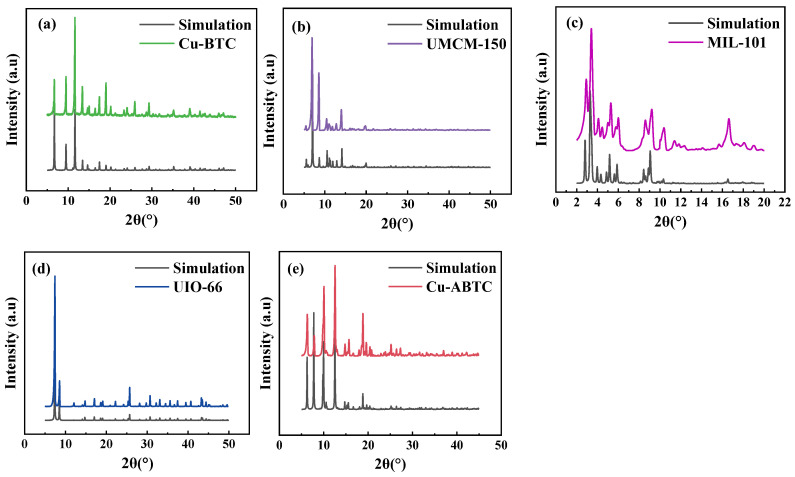
XRD spectra of the five MOFs (**a**) Cu-BTC; (**b**) UMCM-150; (**c**) MIL-101(Cr); (**d**) UIO-66; and (**e**) Cu-ABTC.

XRD spectra of Cu-BTC, UMCM-150, MIL-101(Cr), UIO-66, and Cu-ABTC were shown in Figure 1. The characteristic diffraction peaks of the five MOFs were well matched with the simulated one and literature data [27,28,29], indicating the dedicated MOFs were successfully synthesized. The diffraction peaks of Cu-BTC are at 6.5°, 9.5°, 11.5°, and 13.4°. The diffraction peaks of UMCM-150 are at 6.8°, 8.7°, 10.6°, and 14.1°. The diffraction peaks of MIL-101 are at 2.8°, 3.3°, 5.2°, and 9.0°. The diffraction peaks of UIO-66 are at 7.3°, 8.5°, and 25.7°. The diffraction peaks of Cu-ABTC are at 6.2°, 7.7°, 9.9°, and 12.5°. As shown in Figure 1, the intensities of the characteristic peaks of the five MOFs was weaker than those in the simulation, which may be due to the low crystallinity of the synthesized MOFs. The average crystallite size of Cu-BTC, UMCM-150, MIL-101(Cr), UIO-66, and Cu-ABTC were 976, 371, 68, 364, and >1000 Å, respectively, which may result in the variation of the surface area and pore size of five MOFs and thus impacted the adsorption performance.

**Figure 2:**
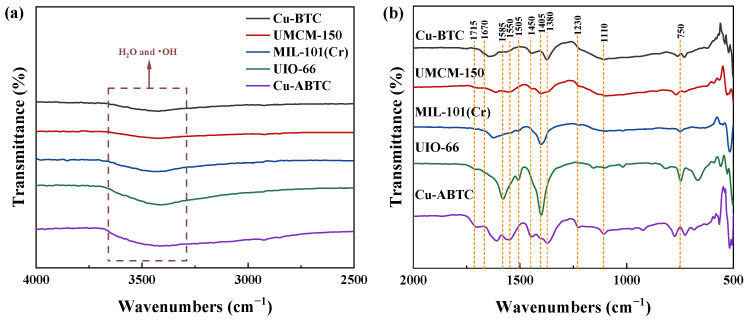
FTIR spectra of the Cu-BTC, UMCM-150, MIL-101(Cr), UIO-66, and Cu-ABTC, (**a**) 2500–4000 cm^−1^; (**b**) 500–2000 cm^−1^.

The FTIR spectra of Cu-BTC, UMCM-150, MIL-101(Cr), UIO-66, and Cu-ABTC were analyzed to reveal thier surface functional groups (Figure 2). As shown in Figure 2, the prepared Cu-based MOFs such as Cu-BTC, UMCM-150, and Cu-ABTC displayed a typical stretching vibration band of Cu-O at about 725 cm^−1^, illustrating the successful introduction of CuO in the MOFs [30]. For the Cu-BTC, the vibration bands at 1643 cm^−1^ and 1373 cm^−1^ were detected and matched with the symmetry and asymmetry COO^−^ of H_3_BTC. Whereas, the COO^−^ band was shifted to 1550 cm^−1^ and 1401 cm^−1^ in UMCM-150. As for Cu-ABTC, the COO^−^ band was detected at 1371 cm^−1^, and the N=N vibration band was observed at 1612 cm^−1^ [31,32]. The vibration bands at 1400 cm^−1^ and 1579–1623 cm^−1^ in MIL-101(Cr) and UIO-66 are attributed to COO^−^. The bands at 672 cm^−1^ and 559 cm^−1^ were assigned to the characteristic stretching vibration of Cr-O and Zr-O in MIL-101(Cr) and UIO-66, respectively. Furthermore, a broad band at 3000–3500 cm^−1^ was observed in all the five MOFs, which can be assigned for H_2_O and OH^−^. The detected oxygen-containing groups can enhance the interaction between the thiophene sulfur and linkers [33], which can contribute to the improvement of thiophene sulfur adsorption.

**Figure 3:**
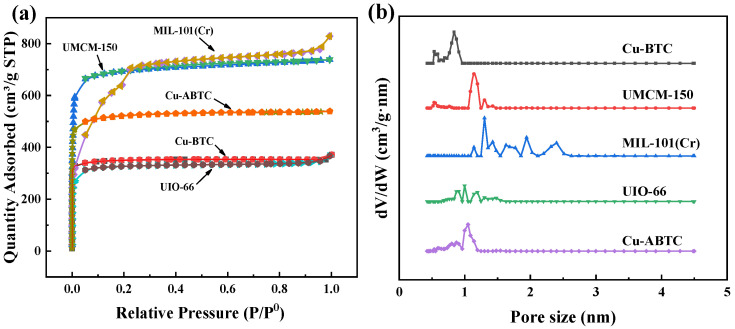
(**a**) Nitrogen adsorption-desorption isotherms and (**b**) NL-DFT pore-size distributions of the Cu-BTC, UMCM-150, MIL-101(Cr), UIO-66, and Cu-ABTC.

As show in Figure 3a, the N_2_ adsorption-desorption isotherms of the five MOFs were measured. All the five MOFs displayed a type I isotherm with a sharp uptake at P/P^0^ < 0.1 and a weak hysteresis loop at P/P^0^ = 0.3–1.0, indicating the domination of micropores in the MOFs [34]. The NL-DFT model was used to further analyze the pore size distribution (Figure 3b). The pore sizes of all the MOFs were mainly located at 1–2 nm. The Cu-BTC, UIO-66, and Cu-ABTC possessed the micropores smaller than 1 nm, which may be helpful to improve the sulfur adsorption [17]. The BET surface area and pore volume of the five MOFs were caculated and listed in Table 1. The order of surface area and pore volumes followed the same trend and can be summarized as follows: MIL-101(Cr) > UMCM-150 > Cu-ABTC > Cu-BTC > UIO-66. The higher the surface area possessed, the greater the pore volume achieved.

### 3.2. Performances of DBT Adsorption

**Figure 4:**
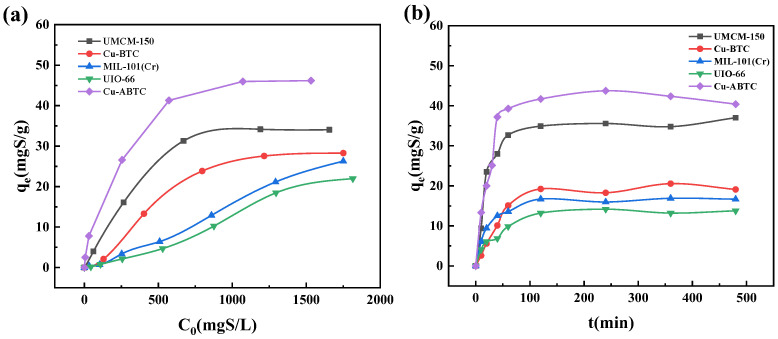
The adsorption performance of the five MOFs (**a**) under various sulfur concentration; (**b**) under different adsorption time. (T = 30 °C, oil-adsorbent ratio = 70 g/g).

The adsorption performances of DBT onto the five MOFs were investigated with a mass ratio of 70 g/g between simulated oil and MOFs at 30 °C. The dosed sulfur concentrations ranged from 10 to 2000 mg S/L. As displayed in Figure 4a, the adsorption capacities of the five MOFs increased with the dosed sulfur concentration. At the same dosed sulfur concentration, the adsorption capacity followed the order of Cu-ABTC > UMCM-150 > Cu-BTC > MIL-101(Cr) > UIO-66, which corresponding adsorption capacities were 46.2, 34.2, 28.3, 26.3, and 22.0 mg S/g, respectively (Figure 4a). For all the five MOFs, the adsorption equilibrium was achieved within 120 min (Figure 4b). The Cu-ABTC exhibited a high adsorption capacity with the highest crystallite size of >1000 Å. MIL-101(Cr) possessing the lowest crystallite size and highest surface area and pore volume (see Table 1) exhibited the lowest adsorption capacity. Cu-BTC with a similar surface area as UIO-66 had nearly twice the absorption quantity of UIO-66.

The three Cu-based MOFs displayed a higher adsorption capacity compared with the MIL-101 and UIO-66. Based on the hard/soft-acid/base principle [35], the sulfur in the DBT belongs to soft base and prefers to interact with Cu-based soft acid rather than the hard acid of Cr-based and Zr-based MOFs [36]. Additionally, the interaction between Cu with empty outermost sorbit and electrons-contained DBT could also enhance the adsorption onto the Cu-based MOFs [11]. Among the three Cu-based MOFs, the adsorption capacity of Cu-BTC was far below that of Cu-ABTC and UMCM-150, which was mainly attributed by its low surface area and pore size. However, the DBT removal by the UMCM-150 with a high surface was inferior to that of Cu-ABTC. It can be explained by the fact that the linker of Cu-ABTC contained not only two benzene rings but also N=N bond, which significantly increased the π-complexation interaction between the MOFs and DBT.

### 3.3. Kinetics of DBT Adsorption

**Figure 5:**
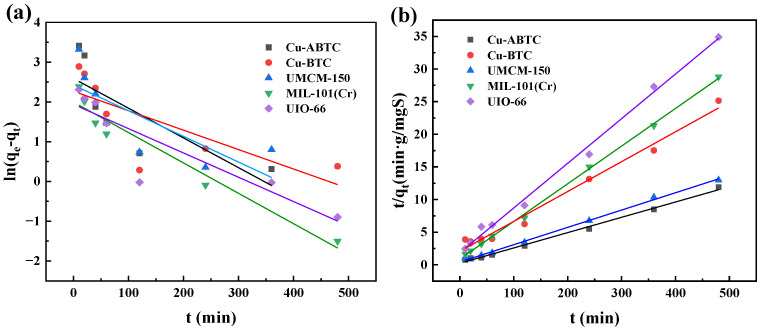
Kinetics of DBT adsorption onto the five MOFs fitted by (**a**) pseudo-first-order and (**b**) pseudo-second-order.

The kinetics of DBT adsorption onto the five MOFs were analyzed by both the pseudo-first-order model and the pseudo-second-order model as shown in Figure 5. The values of kinetic parameters are listed in Table 2. Compared with the pseudo-first-order model, the pseudo-second-order model plots exhibited a wonderful linearity correlation with all the goodness (R^2^) over 0.99. Moreover, the calculated values of q_e,cal_ by pseudo-second-order model exhibited the closest value to the experimental q_e,exp_, indicating that the DBT adsorption kinetics by the five MOFs could be described by the pseudo-second-order model. This result also confirmed that the chemisorption was involved in the DBT adsorption onto the tested MOFs [37]. The order of adsorption rate constant, k_2_, was MIL-101 (Cr) > UIO-66 > Cu ABTC > UMCM-150 > Cu BTC. The MIL-101 (Cr) with the highest adsorption rate constant may be contributed by its largest pore diameter, which reduced the DBT mass transfer limitation and thus enhanced the DBT adsorption [38]. Since the pore size of Cu-ABTC was similar to the DBT, its adsorption rate was limited due to the diffusion resistance.

### 3.4. Adsorption Condition Optimization

**Figure 6:**
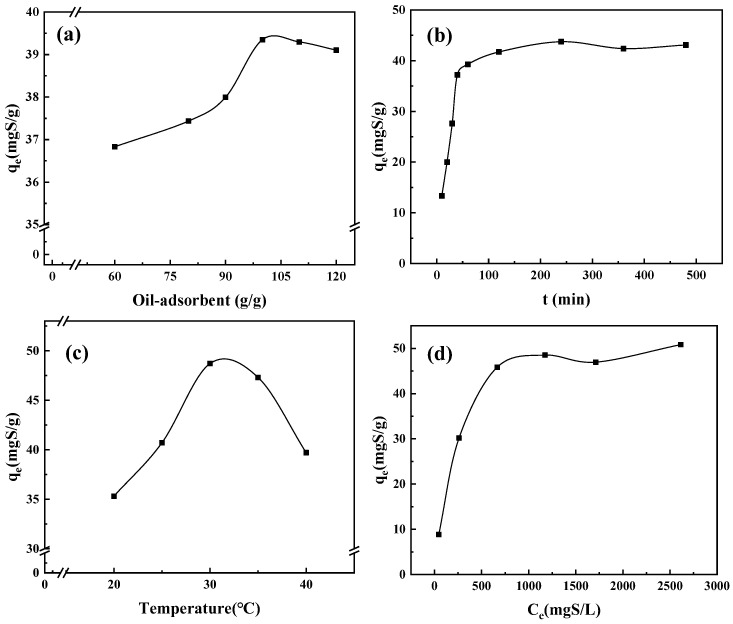
Optimization of adsorption conditions onto Cu-ABTC (**a**) oil-adsorbent ratio; (**b**) adsorption time; (**c**) temperature and (**d**) initial sulfur concentration. (in (**a**–**c**), $C_{0}$ = 1000 mgS/L; in (**a**,**b**,**d**), T = 30 °C; in (**b**–**d**), oil-adsorbent ratio = 100 g/g).

Since the Cu-ABTC exhibited a high DBT adsorption capacity, it was used as the representative adsorbent to optimize the operation conditions. The effects of oil-adsorbent ratio, adsorption time, operation temperature, and the dosed sulfur concentration on the DBT adsorption were investigated. As shown in Figure 6a, the adsorption capacity was increased with the oil-adsorbent ratio ranging from 60 g/g to 100 g/g. When the oil-adsorbent ratio further increased to 120 g/g, the adsorption capacity slightly decreased. Therefore, the oil-adsorbent ratio of 100 g/g was recommended for the DBT adsorption onto the Cu-ABTC. The DBT adsorption onto the Cu-ABTC quickly occurred with a sulfur concentration of 1000 mgS/L and an oil-adsorbent ratio of 100 g/g. ~90% of DBT was adsorbed within 60 min and the adsorption saturation was reached within 250 min (Figure 6b). The recommended adsorption time was 60 min. As shown in Figure 6c, the DBT adsorption efficiency was gradually increased with the temperature rising from 15 to 30 °C and then remarkably decreased from 30 to 40 °C. Typically, a high temperature was not conducive to physical adsorption and thus the DBT adsorption capacity declined [39]. The temperature of 30 °C was regarded as the optimal condition. As shown in Figure 6d, the adsorption capacity was increased with the dosed sulfur concentration. When the dosed sulfur concentration reached 1000 mg S/L, the highest adsorption capacity achieved. Further increase in the dosed sulfur concentration did not boost the adsorption capacity. Overall, the optimized adsorption conditions of DBT onto Cu-ABTC were a temperature of 30 °C, an adsorption time of 60 min, an oil-adsorbent ratio of 100 g/g, and a sulfur concentration of 1000 mg S/L.

Thermodynamic parameters such as $\Delta G^{o}$, $\Delta H^{o}$, and $\Delta S^{o}$ of the DBT adsorption onto Cu-ABTC were calculated and listed in Table 3. The positive values of $\Delta G^{o}$ at 20–40 °C demonstrated the feasibility and spontaneous nature of the DBT adsorption onto Cu-ABTC. The amount of $\Delta G^{o}$ decreased with the increasing temperature, indicating that the adsorption of DBT is positively correlated with the temperature. The value of $\Delta H^{o}$ is positive and smaller than 42 kJ/mol, suggesting that the reaction is endothermic. The positive value of $\Delta S^{o}$ indicates that the adsorption is irreversible [40].

## 4. Conclusions

In summary, the five MOFs with Cu, Cr and Zr as metal centers were successfully synthesized and proven by the evidence of XRD and FRIT spectra. The adsorption test revealed that the three Cu-based MOFs, such as Cu-ABTC, UMCM-150 and Cu-BTC, exhibted a high DBT adsorption performance compared with the Cr-based MIL-101 and Zr-based UIO-66. It was confirmed that the adsorption of DBT onto the MOFs not only depended on the physisorption pertained to the surface area and pore volume but also relied on the chemisorption by the metal center and organic linkers of the MOFs. Additionally, the pseudo second-order kinetics exhibited a good correlation with the experimental values. Overall, this work can provide some guidance to the design of efficient MOFs for deep desulfurization from the fuel oil.

## Figures and Tables

**Table 1:** BET surface area and pore volumes of the five MOFs.

MOFs	S_BET_ (m^2^/g)	V_total_ (cm^3^/g)
MIL-101(Cr)	2413.76	1.19
UMCM-150	2114.34	1.14
Cu-ABTC	1588.49	0.83
Cu-BTC	1078.95	0.55
UIO-66	993.96	0.53

**Table 2:** Parameters of the kinetic model.

MOFs		Pseudo-First-Order			Pseudo-Second-Order		
	q_e,exp_ (mgS/g)	q_e,cal_ (mgS/g)	k_1_ (min^−1^)	R^2^	q_e,cal_ (mgS/g)	k_2_ g/(mgS·min)	R^2^
Cu-ABTC	43.74	13.32	0.0075	0.5191	42.55	0.0024	0.9947
Cu-BTC	20.55	9.65	0.0049	0.4844	21.83	0.0010	0.9824
UMCM-150	37.00	11.18	0.0065	0.5156	37.74	0.0017	0.9970
MIL-101(Cr)	16.89	7.34	0.0076	0.9406	17.33	0.0038	0.9989
UIO-66	14.16	6.95	0.0061	0.7632	14.62	0.0025	0.9943

**Table 3:** Thermodynamic parameters for the adsorption of DBT on Cu-ABTC.

$\Delta H^{o}\;(\mathrm{kJ}/\mathrm{mol})$	$\Delta S^{o}\;(J/\mathrm{mol}.K)$	$\Delta G^{o}\;(\mathrm{kJ}/\mathrm{mol})$		
		293K	298K	303K
34.067	145.328	−8.527	−9.211	−9.981

## Data Availability

The data presented in this study are available on request from the corresponding author. The data are not publicly available due to privacy.

## References

[1] Srivastava V.C. (2012). An evaluation of desulfurization technologies for sulfur removal from liquid fuels. RSC Adv..

[2] Lu P., Yin M., Chen J., Wang Q., Ye C., Qiu T. (2022). ZiF-8-derived P, N-co-doped hierarchical carbon: Synergistic and high-efficiency desulfurization adsorbents. Chem. Eng. J..

[3] Wang R., Yu F., Zhang G., Zhao H. (2010). Performance evaluation of the carbon nanotubes supported Cs_2.5_H_0.5_PW_12_O_40_ as efficient and recoverable catalyst for the oxidative removal of dibenzothiophene. Catal. Today.

[4] Saha B., Vedachalam S., Dalai A.K. (2021). Review on recent advances in adsorptive desulfurization. Fuel Process. Technol..

[5] Zhang Y.H., Liu M.Q., Liu M.M., Zhao L., Gao J.S., Xu C.M., Gao X.H., Liu X.Q. (2021). Fundamental research on the influence of mercaptan and thioether structure on the solvent extraction of fluid catalytic cracking naphtha. Sep. Purif. Technol..

[6] Haruna A., Merican Z.M.A., Musa S.G. (2022). Recent advances in catalytic oxidative desulfurization of fuel oil—A review. J. Ind. Eng. Chem..

[7] Haruna A., Merican A.M.Z., Gani M.S., Abubakar S. (2022). Sulfur removal technologies from fuel oil for safe and sustainable environment. Fuel.

[8] Abro R., Abdeltawab A.A., Al-Deyab S.S., Yu G., Qazi A.B., Gao S., Chen X. (2014). A review of extractive desulfurization of fuel oils using ionic liquids. RSC Adv..

[9] Soleimani M., Bassi A., Margaritis A. (2007). Biodesulfurization of refractory organic sulfur compounds in fossil fuels. Biotechnol. Adv..

[10] Luna M.D.G., Samaniego M.L., Ong D.C., Wan M.W., Lu M.C. (2018). Kinetics of sulfur removal in high shear mixing-assisted oxidative-adsorptive desulfurization of diesel. J. Clean. Prod..

[11] Tran D.T., Palomino J.M., Oliver S.R.J. (2018). Desulfurization of JP-8 jet fuel: Challenges and adsorptive materials. RSC Adv..

[12] Zheng M., Chen J., Zhang L., Cheng Y., Lu C., Liu Y., Singh A., Trivedi M., Kumar A., Liu J. (2022). Metal organic frameworks as efficient adsorbents for drugs from wastewater. Mater. Today. Commum..

[13] Zhong Y.Y., Chen C., Liu S., Lu C.Y., Liu D., Pan Y., Sakiyama H., Muddassir M., Liu J.Q. (2021). A new magnetic adsorbent of eggshell-zeolitic imidazolate framework for highly efficient removal of norfloxacin. Dalton Trans..

[14] Zhou S.H., Lu L., Liu D., Wang J., Sakiyama H., Muddassir M., Nezamzadeh A., Liu J.Q. (2021). Series of highly stable Cd_(II)_-based MOFs as sensitive and selective sensors for detection of nitrofuran antibiotic. CrystEngComm.

[15] Pan Y., Rao C.Y., Tan X.L., Ling Y., Singh A., Kumar A., Li B.H., Liu J.Q. (2022). Cobalt-seamed C-methylpyrogallol 4 arene nanocapsules-derived magnetic carbon cubes as advanced adsorbent toward drug contaminant removal. Chem. Eng. J..

[16] Zhang Y., Li G., Kong L., Lu H. (2018). Deep oxidative desulfurization catalyzed by Ti-based metal-organic frameworks. Fuel.

[17] Cychosz K.A., Wong-Foy A.G., Matzger A.J. (2008). Liquid phase adsorption by microporous coordination polymers: Removal of organosulfur compounds. J. Am. Chem. Soc..

[18] Khan N.A., Jhung S.H. (2012). Low-temperature loading of Cu^+^ species over porous metal-organic frameworks (MOFs) and adsorptive desulfurization with Cu^+^-loaded MOFs. J. Hazard. Mater..

[19] Huang M., Chang G., Su Y., Xing H., Zhang Z., Yang Y., Ren Q., Bao Z., Chen B. (2015). A metal-organic framework with immobilized Ag(i) for highly efficient desulfurization of liquid fuels. Chem. Commun..

[20] Shi F., Hammoud M., Thompson L.T. (2011). Selective adsorption of dibenzothiophene by functionalized metal organic framework sorbents. Appl. Catal. B.

[21] Li J., Yang Z., Hu G., Zhao J. (2020). Heteropolyacid supported MOF fibers for oxidative desulfurization of fuel. Chem. Eng. J..

[22] Mguni L.L., Yao Y., Ren J., Liu X., Hildebrandt D. (2021). Modulated synthesized Ni-based MOF with improved adsorptive desulfurization activity. J. Clean. Prod..

[23] Chu L., Guo J., Wang L., Liu H., Yan J., Wu L., Yang M., Wang G. (2022). Synthesis of defected UIO-66 with boosting the catalytic performance via rapid crystallization. Appl. Organomet. Chem..

[24] Vakili R., Gholami R., Stere C.E., Chansai S., Fan X. (2019). Plasma-assisted catalytic dry reforming of methane (DRM) over metal-organic frameworks (MOFs)-based catalysts. Appl. Catal. B Environ..

[25] Wang S., Liu J., Pulido B., Li Y., Mahalingam D., Nunes S.P. (2020). Oriented zeolitic imidazolate framework (ZIF) nanocrystal films for molecular separation membranes. ACS Appl. Nano Mater..

[26] Huo Q., Li J., Qi X., Liu G., Zhang X., Zhang B., Ning Y., Fu Y., Liu J., Liu S. (2019). Cu, Zn-embedded MOF-derived bimetallic porous carbon for adsorption desulfurization. Chem. Eng. J..

[27] Wong-Foy A.G., Lebel O., Matzger A.J. (2007). Porous crystal derived from a tricarboxylate linker with two distinct binding motifs. J. Am. Chem. Soc..

[28] Qiu J., Feng Y., Zhang X., Jia M., Yao J. (2017). Acid-promoted synthesis of UiO-66 for highly selective adsorption of anionic dyes: Adsorption performance and mechanisms. J. Colloid Interface Sci..

[29] Xue M., Zhu G.S., Li Y.X., Zhao X.J., Jin Z., Kang E., Qiu S.L. (2008). Structure, hydrogen storage, and luminescence properties of three 3D metal-organic frameworks with NbO and PtS topologies. Cryst. Growth Des..

[30] Hu X., Liu X., Zhang X., Chai H., Huang Y. (2018). One-pot synthesis of the CuNCs/ZIF-8 nanocomposites for sensitively detecting H_2_O_2_ and screening of oxidase activity. Biosens. Bioelectron..

[31] Qin L., Liang F.L., Li Y., Wu J.A., Guan S.Y., Wu M.Y., Xie S.L., Luo M.S., Ma D.Y. (2022). A 2D porous zinc-organic framework platform for loading of 5-fluorouracil. Inorganics.

[32] Qin L., Li Y., Liang F., Li L., Lan Y., Li Z., Lu X., Yang M., Ma D. (2022). A microporous 2D cobalt-based MOF with pyridyl sites and open metal sites for selective adsorption of CO_2_. Microporous Mesoporous Mater..

[33] Deng L., Lu B., Li J., Lv G., Du S., Shi J., Yang Y. (2017). Effect of pore structure and oxygen-containing groups on adsorption of dibenzothiophene over activated carbon. Fuel.

[34] Huang M., Mi K., Zhang J., Liu H., Yu T., Yuan A., Kong Q., Xiong S. (2017). MOF-derived bi-metal embedded N-doped carbon polyhedral nanocages with enhanced lithium storage. J. Mater..

[35] Aliyu M., Azhar A., Arief C. (2020). Cation exchange in metal-organic frameworks (MOFs): The hard-soft acid-base (HSAB) principle appraisal. Inorg. Chim. Acta.

[36] Zheng S., Sun Y., Xue H., Braunstein P., Huang W., Pang H. (2022). Dual-ligand and hard-soft-acid-base strategies to optimize metal-organic framework nanocrystals for stable electrochemical cycling performance. Natl. Sci. Rev..

[37] Saleh T.A., Danmaliki G.I. (2016). Adsorptive desulfurization of dibenzothiophene from fuels by rubber tyres-derived carbons: Kinetics and isotherms evaluation. Process. Saf. Environ. Prot..

[38] Li X., Mao Y., Leng K., Ye G., Sun Y., Xu W. (2017). Enhancement of oxidative desulfurization performance over amorphous titania by doping MIL-101(Cr). Microporous Mesoporous Mater..

[39] Liu J., Li X.M., He J., Wang L.Y., Lei J.D. (2018). Combining the photocatalysis and absorption properties of core-shell Cu-BTC@TiO_2_ microspheres: Highly efficient desulfurization of thiophenic compounds from fuel. Materials.

[40] Yc A., Jyl B., Djlab C. (2016). Thermodynamic parameters for adsorption equilibrium of heavy metals and dyes from wastewaters: Research updated—Sciencedirect. Bioresour. Technol..

